# Role of Long Non-Coding RNAs in Conferring Resistance in Tumors of the Nervous System

**DOI:** 10.3389/fonc.2021.670917

**Published:** 2021-06-09

**Authors:** Soudeh Ghafouri-Fard, Amin Agabalazadeh, Atefe Abak, Hamed Shoorei, Mohammad Mehdi Hassanzadeh Taheri, Mohammad Taheri, Guive Sharifi

**Affiliations:** ^1^ Department of Medical Genetics, Shahid Beheshti University of Medical Sciences, Tehran, Iran; ^2^ Department of Pharmacology, Tabriz University of Medical Sciences, Tabriz, Iran; ^3^ Department of Medical Genetics, Faculty of Medicine, Tabriz University of Medical Sciences, Tabriz, Iran; ^4^ Department of Anatomical Sciences, Faculty of Medicine, Birjand University of Medical Sciences, Birjand, Iran; ^5^ Urology and Nephrology Research Center, Shahid Beheshti University of Medical Sciences, Tehran, Iran; ^6^ Skull Base Research Center, Loghman Hakim Hospital, Shahid Beheshti University of Medical Sciences, Tehran, Iran

**Keywords:** long non-coding RNA, lncRNA, chemoresistance, brain tumor, expression

## Abstract

Tumors of the nervous system can be originated from several locations. They mostly have high mortality and morbidity rate. The emergence of resistance to chemotherapeutic agents is a hurdle in the treatment of patients. Long non-coding RNAs (lncRNAs) have been shown to influence the response of glioblastoma/glioma and neuroblastoma to chemotherapeutic agents. MALAT1, NEAT1, and H19 are among lncRNAs that affect the response of glioma/glioblastoma to chemotherapy. As well as that, NORAD, SNHG7, and SNHG16 have been shown to be involved in conferring this phenotype in neuroblastoma. Prior identification of expression amounts of certain lncRNAs would help in the better design of therapeutic regimens. In the current manuscript, we summarize the impact of lncRNAs on chemoresistance in glioma/glioblastoma and neuroblastoma.

## Introduction

Tumors of the nervous system can be originated from several cellular compartments. The main classes of these tumors are glioma, meningioma, neuroblastoma, and spinal tumors (1). Although being quite rare, brain tumors are considered as high mortality cancers (2). Their protected position in the brain makes these neoplasms difficult to cure. Surgical removal of the tumor, radiotherapy, and chemotherapy are currently available therapeutic options for brain tumors. However, these options are associated with possible permanent morbidity for patients and incomplete cure of cancer (2). Inherent or attained chemoresistance is the chief reason for treatment failure in these patients (3). Alkylating agents constitute the backbone of chemotherapeutic regimens for brain tumors. These agents induce DNA damage and consequently activate apoptosis, yet their efficiency in killing cancer cells depends on the DNA repair system (3). As an example of an orally bioavailable alkylating agent, temozolomide (TMZ) has been used widely in the treatment of patients with brain tumors. This agent is spontaneously transformed to its active metabolite 5-(3-methyl triazen-1-yl) imidazole-4-carboxamide (MTIC) without requiring hepatic activation.). Furthermore, TMZ is an effective radiosensitizer and a vital constituent of chemoradiotherapy for patients with newly-diagnosed glioblastoma (4). Resistance to TMZ has been detected in about half of patients. Up-regulation of O6-methylguanine methyltransferase (MGMT) and defects in the DNA repair pathway are among possible mechanisms for resistance to this agent (5). Another recently acknowledged cause of chemoresistance in tumors of the nervous system and related cell lines is the aberrant expression of long non-coding RNAs (lncRNAs). In the current manuscript, we summarize the impact of lncRNAs on chemoresistance in glioma/glioblastoma and neuroblastoma.

## LncRNAs Functions

**Figure 1:**
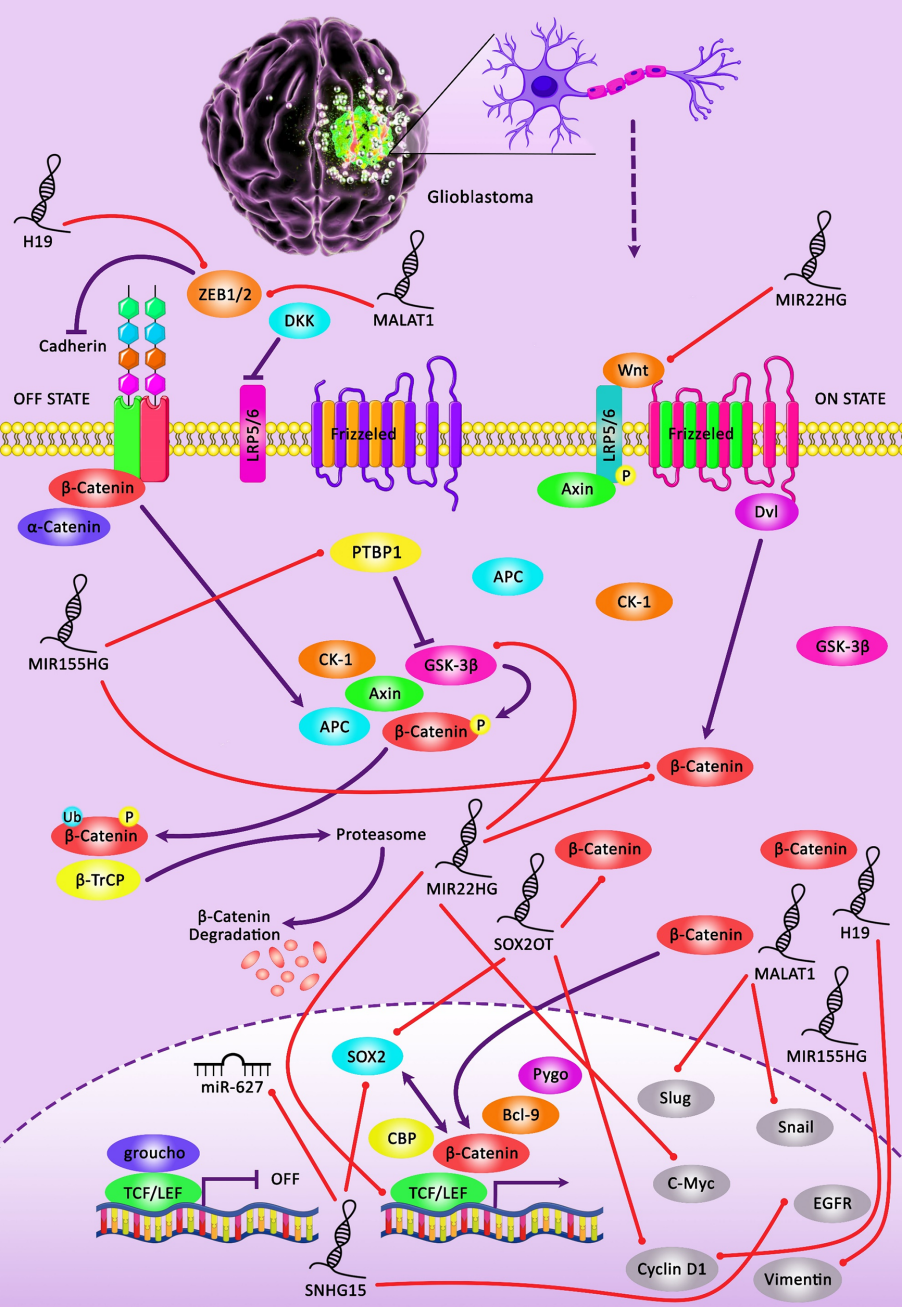
A schematic illustration of the crosstalk between lncRNAs and Wnt/β-catenin pathway involved in the modulation of the sensitivity of glioma cells to chemotherapeutic agents. Downregulation of lncRNA H19 could promote the sensitivity of glioma cells to temozolomide *via* inhibiting EMT through the suppression of the Wnt/β-Catenin signaling cascade. Silencing of H19 could downregulate the expression level of β-catenin and its downstream targets c-myc and Survivin in temozolomide-treated glioma cells (11). Besides, downregulating the expression of lncRNA MIR22HG could suppress the Wnt/β-catenin signaling pathway *via* loss of miR-22-3p and -5p. This could in turn lead to attenuating cell proliferation, invasion as well as tumor growth in glioma cells. MIR22HG silencing could result in downregulating the expression level of β-catenin, a key transcriptional regulator of Wnt, along with the inhibition of several Wnt downstream targets, containing c-Myc, cyclin D1, and LEF1, as well as a reduction in the expression of phospho-GSK3β (Ser9) in tumor cells (12). Besides, upregulation of lncRNA MIR155HG could promote temozolomide resistance in glioma cells through directly regulating canonical Wnt/β-catenin pathway activation *via* binding to PTBP1 in tumor cells (13).

Novel sequencing methods have enabled comprehensive genomic and transcriptomic analyses and shown transcription of a total of 85% of the human genome (6, 7). Based on the results of ENCODE projects, most human transcriptomes are non-coding RNAs (8). LncRNAs with sizes of more than 200 nucleotides constitute a major part of the transcriptome. These transcripts are considered essential regulators of gene transcription. Their functions as signals, decoys, scaffolds, guide transcripts, and enhancers have endowed them the aptitude to control gene expression *via* different routes. Through having “decoy” binding sites, they can sequester transcription factors, catalytic molecules, constituents of chromatin remodeling complexes, and microRNAs (miRNAs), thus decreasing their bioavailability (9). Dysregulation of lncRNAs has been noted in tumors of the nervous system (10). **Figure 1** indicates the role of several lncRNAs in modulating the sensitivity of tumor cells to various chemotherapeutic agents *via* regulating the Wnt-β-catenin signaling pathway which is a highly conserved cascade and is activated in the development of glioma cells. Wnt/β-catenin signaling is an evolutionary conserved axis that controls important cellular functions, namely proliferation, differentiation, migratory potential, genetic stability, cell death and renewal of stem cells, thus it has important roles in the carcinogenesis (14).

## LncRNAs and Chemoresistance in Glioma/Glioblastoma

Abnormal expression of several lncRNAs has been detected in resistant glioma/glioblastoma tumors or related cell lines. MALAT1, NEAT1, H19, and HOTAIR are among the mostly assessed lncRNAs in this field.

### MALAT1

Li et al. have reported higher levels of MALAT1 in the U251/TMZ and U87/TMZ cells compared with the parental lines. Small interfering (si)RNA-mediated MALAT1 silencing has down-regulated expressions of MDR1, MRP5, and LRP1, increased sensitivity to TMZ, and decreased ZEB1 levels. *In vivo* experiments have also verified the impact of MALAT1 up-regulation in conferring TMZ resistance and upregulating ZEB1 levels. Taken together, MALAT1 can enhance the resistance of glioma cells to TMZ through regulating ZEB1 (15). Vassallo et al. have shown that MALAT1 silencing decreases migration of glioblastoma cells, without affecting proliferation. Meanwhile, down-regulation of WIF1 has been shown to enhance the migratory aptitude of glioblastoma *via* WNT5A that induces expression of MALAT1. They have suggested the contribution of canonical and non-canonical WNT routes in the pathogenesis of glioblastoma (16). Moreover, MALAT1 has been reported to induce chemoresistance to TMZ through suppressing miR-203 expression and promoting the expression of thymidylate synthase (17). Similarly, Cai et al. have reported up-regulation of MALAT1 in TMZ-resistant glioblastoma cells. MALAT1 silencing has reduced TMZ resistance of these cells as documented in cell lines and animal models. Functionally, MALAT1 confers this phenotype by inhibiting the miR-101 signaling pathway in glioblastoma cells (18). A clinical study in this field has shown the association between elevated serum levels of MALAT1 and poor response to TMZ and low survival rate of patients with glioblastoma (17). Notably, functional studies have verified that MALAT1 silencing reverses TMZ resistance in glioblastoma cell lines. MALAT1 exerts its function through modulating the expression of miR-203, thus reducing thymidylate synthase (TS) levels (17). Finally, Voce et al. have assessed the expression profile of glioblastoma cells to detect NF-κB-related transcripts whose expressions are changed following TMZ treatment. MALAT1 has been identified as one of the utmost elevated transcripts. Moreover, expression of MALAT1 has been simultaneously regulated by p50 and p53. TMZ has been shown to inhibit p50 recruitment to its cognate element following phosphorylation of Ser329. Administration of anti-MALAT1 siRNA *via* nanoparticles has enhanced response to TMZ in xenograft models of glioblastoma (19).

### NEAT1

Expression of NEAT1 has been found to be elevated in serum samples of glioblastoma patients and glioma stem cells isolated from related cell lines. NEAT1 silencing has inhibited the malignant behaviors of these cells, as has been evident by the reduction of their proliferation, migration, and invasion. Functional studies have verified let-7g-5p as a target of NEAT1. Expression of MAP3K1, as a target of let-7g-5p, has been enhanced by NEAT1, Therefore, NEAT1 enhances malignant features of glioma stem cell and chemoresistant phenotype *via* let-7g-5p/MAP3K1 axis (20). Similarly, expression of NEAT1 has been lower in the TMZ-sensitive glioblastoma tissues and cell lines compared with TMZ-resistant ones. NEAT1 silencing has remarkably promoted TMZ-associated cell apoptosis in glioblastoma cells. Consistently, MGMT levels have been higher in TMZ-resistant cell lines. NEAT1 silencing has decreased mRNA and protein levels of MGMT (21).

### H19

H19 is another oncogenic lncRNA in glioblastoma whose expression has been correlated with the expression of numerous genes participating in the growth and progression of this neoplasm. H19 silencing has reduced *via*bility, migratory potential, and invasiveness of glioblastoma cells. Notably, H19 expression is inversely correlated with the expression of NKD1, an inhibitor of the Wnt pathway, thus H19 may modulate NKD1 expression *via* EZH2-associated H3K27 trimethylation. H19 binding with EZH2 has been verified in glioblastoma cells (22). H19 silencing has been shown to enhance TMZ cytotoxicity in glioma cells through inhibiting epithelial-mesenchymal transition (EMT) *via* the Wnt/β-catenin pathway (11) and inactivating NF-kB signaling (23).

### HOTAIR

Expression of HOTAIR has been elevated in TMZ-resistant glioblastoma cells and its silencing has suppressed proliferation, migration, invasion, and EMT in TMZ-resistant cells. Notably, exosomal transfer of this lncRNA HOTAIR has conferred TMZ resistance *via* modulating miR-519a-3p/RRM1 molecular route (24). HOTAIR silencing has also decreased HK2 expression, thus suppressing cell proliferation and enhancing sensitivity to TMZ both *in vivo* and *in vitro*. HOTAIR increases HK2 levels by influencing miR-125 levels, which suppresses cell proliferation and increases TMZ-associated cell death (25).

### Other lncRNAs

Several other lncRNAs have also been shown to affect the response of glioblastoma/glioma cells to therapeutic agents. Some lncRNAs affect autophagy. Autophagy is a fundamental capability of cells to reinstate the energy equilibrium throughput the periods of fluctuating nutrient accessibility (26). During this evolutionarily conserved process, impaired or useless biomolecules, organelles, or other cytoplasmic elements are transferred to the lysosomal system be targeted for degradation (27). Dysregulation of autophagy is linked with tumorigenesis and resistance of cancer cells to therapeutics (28).

**Figure 2:**
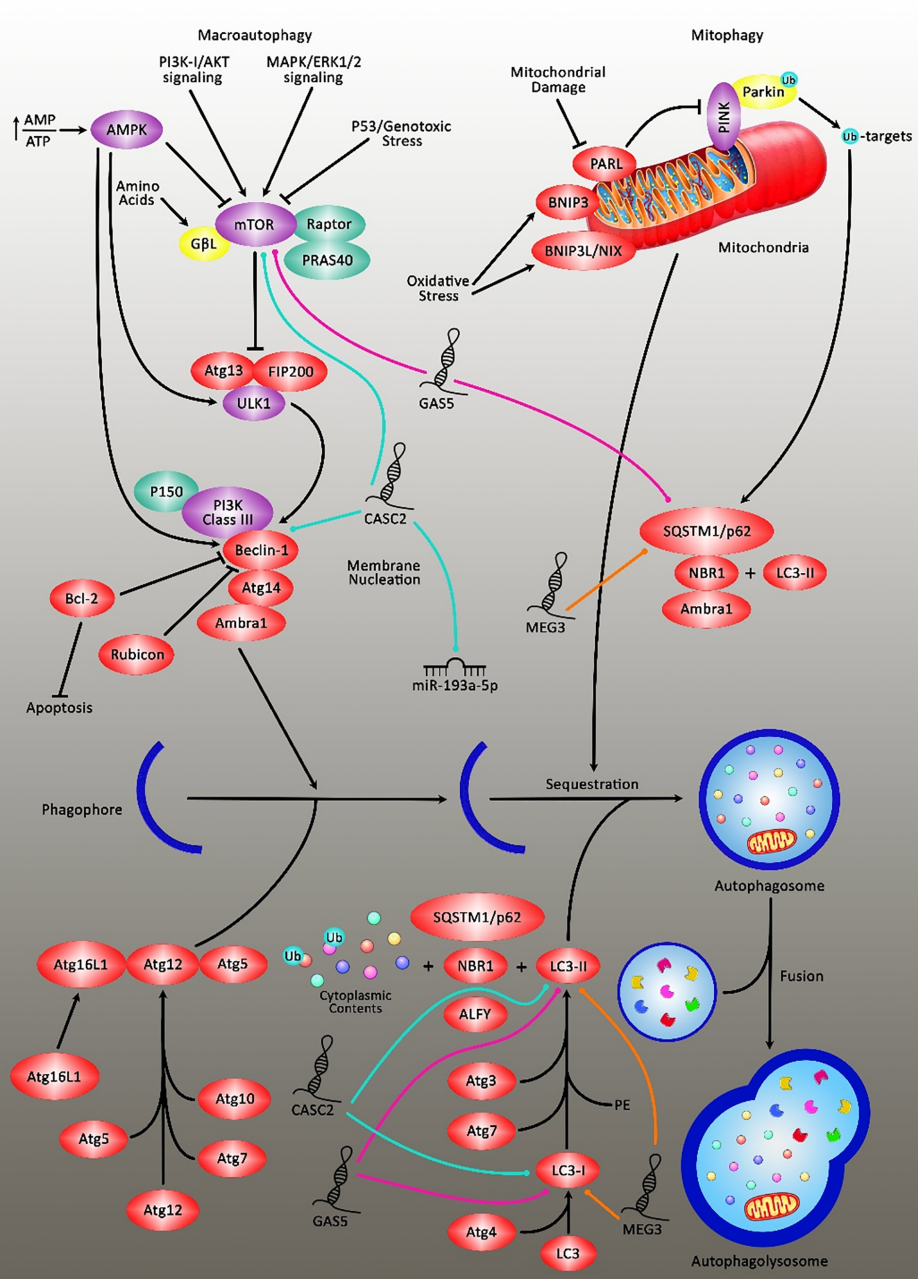
A schematic diagram of the inhibition of autophagy cascade *via* long noncoding RNAs in glioblastoma in an mTOR‐dependent manner. Overexpression of lncRNA CASC2 could downregulate the expression level of miR-193a-5p, which could, in turn, lead to reducing temozolomide-induced autophagy and promoting cell death through suppressing the expression level of mTOR, and thereby resulting in enhancing the sensitivity of glioma cells to temozolomide cytotoxicity to the large extent (55). Furthermore, the elevation of lncRNA GAS5 could enhance glioma cell sensitivity to cisplatin. Cisplatin could evoke excessive autophagy concomitant *via* promoting and suppressing the expression levels of LC3II and p62 respectively, which was negatively inhibited after GAS5 overexpression. Therefore, GAS5 could attenuate the resistance of glioma cells to cisplatin by restraining excessive autophagy through the activation of mTOR signaling (54). Also, upregulation of MEG3 could eliminate cisplatin-induced autophagy in glioma cells *via* directly targeting LC3II and p62 in tumor cells. The suppression of autophagy or knockdown of ATG5 could reverse the reduction in cell apoptosis caused by MEG3 knockdown in glioma cells treated with cisplatin (30).

Expression of TUSC7 has been decreased in TMZ-resistant glioblastoma cells and tissues. Ectopic expression of TUSC7 has inhibited TMZ resistance and decreased expression of MDR1. TUSC7 exerts its function by suppressing miR-10a levels (29). Ma et al. have reported over-expression of MEG3 in glioma cells treated with cisplatin. Up-regulation of MEG3 has increased the sensitivity of glioblastoma cells to cisplatin. Functionally, MEG3 attenuates cisplatin-induced autophagy (30). In a high throughput study, Zeng et al. have compared the expression of mRNAs and lncRNAs between a TMZ-resistant glioblastoma cell line and parental cells. They have reported differential expression of more than 2000 lncRNAs between these cells. Notably, the ECM−receptor interaction pathway has been downregulated and ECM-related collagen I, fibronectin, laminin, and CD44 have been correlated with resistance phenotype *in vitro* (31). **Table 1** shows the list of lncRNAs that modulate the response of glioblastoma/glioma to chemotherapy. **Figure 2** demonstrates the role of various long noncoding RNAs including CASC2 and GAS5 in suppressing the autophagy pathway through regulating mTOR expression in glioma cells.

**Table 1:** LncRNAs that modulate the response of glioblastoma/glioma to chemotherapy.

lncRNA	Samples	Cell Lines	Target/pathway	Function	Kaplan Meier Analysis	Ref
RP11-838N2.4	–	U87, U251, U87/TMZ, U251/TMZ,	miR-10a, TGFB 1, TGFBR1, Smad-2/3/4	RP11-838N2.4 by inhibiting the functions of miR-10a could increase temozolomide cytotoxic effect in GBM.	–	(32)
MALAT1	Mouse	U251, U87, U251/TMZU87/TMZ	ZEB1, Snail, SLUG	MALAT1 by regulating ZEB1 could decrease the sensitivity of resistant GBM.	–	(15)
MALAT1	Mouse	LN-229, LN-428,LN-319, LN-18,	p-MKK3/6, p-p38,P-ERK, WNT/Ca^2+^	WIF1 could increase the migratory possibility of GBM *via* WNT5A that activates the WNT/Ca^2+^ pathway and MALAT1.	–	(16)
MALAT1	Human	U87, U251, U87/TMZ, U251/TMZ	miR-203, TS	MALAT1 could induce chemoresistance to TMZ by suppressing miR-203 and promoting thymidylate synthase expression.	–	(17)
MALAT1	Human	U251, U251/TMZ	miR-101, MRP1, MGMT, p-gp	Knockdown of MALAT1 by promoting miR-101 could inhibit resistance to TMZ.	–	(18)
MALAT1	Human,Mouse	U87, T98G, LN-18,U87/TMZ, T98G/TMZ, LN-18/TMZ	AERG, CCL2, CXCL4	MALAT1 silencing could sensitize glioblastoma to TMZ.	–	(33)
MALAT1		U87, A172, U251, U87/TMZ,A172/TMZ, U87/TMZ	p53, NF-kB	p50 and p52 are primary regulators of this ncRNA.	–	(19)
TUSC7	Human	U87, U87TMZ	miR-10a, MDR1	TUSC7 by targeting miR-10a could inhibit TMZ resistance in GBM.	–	(29)
NEAT1	Human	U87, U251, U87/TMZ, U251/TMZ	let-7g-5p, MAP3K1, E-cadherin, N-cadherin	NEAT1 by regulating the let-7g-5p/MAP3K1 axis could promote malignant phenotypes and TMZ resistance in GBM.	–	(20)
NEAT1	Human	U87, U87/R, U251, U251/R	MGMT	NEAT1 by regulating MGMT could be involved in TMZ resistance in GBM multiforme.	–	(21)
H19	Human	A172, LN229, U87MG, LN18, T98G	NKD1	H19 could contribute to NKD1 repression *via* the recruitment of EZH2 on its promoter.	–	(22)
H19	Human	U87, U251 U87/TMZ U251/TMZ	PARP, MDR, MRP, ABCG2	Knockdown of H19 could enhance the sensitivity of human glioma cells to TMZ.	–	(34)
H19	–	U251, LN229 U251/TMZ LN229/TMZ	Caspase-3, NF-kB	H19 By activating NF-kB signaling could confer TMZ resistance in glioma.	–	(23)
H19	–	U-251, M059J, U251/TMZ, M059J/TMZ	Wnt/β-catenin, Vimentin,ZEB1, c-myc,E-cadherin, Survivin	H19 silencing by suppressing EMT *via* the Wnt/β-catenin pathway could reduce the resistance of human glioma cells to TMZ.	–	(11)
UCA1	Human	U251,U87MG	CXCL4, miR-182, PFKFB2	UCA1/miR-182/PFKFB2 axis could modify GBM-associated stromal cells-mediated glycolysis and invasion of glioma cells.	–	(35)
uc003iax.2, ENST00000443252	Human	U87, U251, U87/TMZ, U251/TMZ	IL-18, DPP4, ABCB1, TP53, Collagen I, Fibronectin, Laminin	Dysregulated lncRNAs could be involved as novel targets so as to overcome acquired TMZ resistance in GBM chemotherapy.	–	(31)
AC023115.3	Human	U87MG,U251MG	PARP,Caspase-3	AC023115.3 could suppress the chemoresistance of GBM by decreasing autophagy.	–	(36)
AC003092.1	Human	U87, U251, U87/TMZ, U251/251	TFPI-2,miR-195	AC003092.1 could help TMZ chemosensitivity *via* the miR-195/TFPI-2 axis modulation in GBM.	–	(37)
TP73-AS1	Human	G26, G7, G26/TMZG7/TMZ	ALDH1A1	TP73-AS1 is involved in aggressiveness and could promote TMZ resistance in GBM cancer stem cells.	–	(38)
ADAMTS9-AS2	Human	T98G, U118, T98G/TMZU118/TMZ	FUS/MDM2, Tubulin	ADAMTS9-AS2 by upregulating the FUS/MDM2 ubiquitination axis could help TMZ resistance in GBM.	–	(39)
SNHG15	Human	HMC3, HMC3/TMZ	miR-627, EGFR, CDK6, Sox2, β-catenin	Modulating SNHG15/CDK6/miR-627 axis by palbocicli could reduce M2-polarization of glioma-associated microglia in GBM multiforme and finally could overcome TMZ resistance.	–	(40)
SNHG12	Mouse	N3S, N3T3rd, U251, U251T3rd	PARP, Caspase-3, RB, CDK4, CDK6, Cyclin-D1, P-MEK, DNMT1, DNMT3a, DNMT3b, MAPK1, E2F7, P-ERK1/2	Knockdown of SNHG12 by increasing MAPK1 and E2F7 expression and activating the MAPK-ERK could restore TMZ sensitivity in GBM.	SNHG12 expression is associated with poor prognosis in GBM.	(41)
NONHSAT163779	Human	U87, U87/TMZ	hsa_circ_0043949, MDR1, MRP1, BCRP, MGMT	NONHSAT163779 and hsa_circ_0043949 could be involved as prognostic biomarkers for the treatment of GBM.	–	(42)
SBF2-AS1	Human	U87, LN229, A172, T98, U251	XRCC4, y-H2AX, Pro-caspase-3, Cleaved-caspase 3	Knockdown of SBF2-AS1 could increase sensitivity to TMZ in GBM.	SBF2-AS1 expression is associated with poor prognosis in GBM.	(43)
OKN-007	Rat	U138, LN18, T98, U251	TGFβ1	OKN-007 could enhance TMZ sensitivity and suppresses TMZ-resistant GBM.	–	(44)
SOX2OT	Human	U87, U251, U87/TMZ, U251/TMZ	MDR1, BCRP1, MRP1, SOX2, ALKBH5, TCF1, Caspase-3/7/8/9, Wnt5a/β-catenin, Cyclin-D1, C-Myc, LEF1	SOX2OT by elevating SOX2 expression *via* ALKBH5-mediated epigenetic regulation could promote TMZ resistance.	Elevated SOX2OT expression is associated with poor prognosis in GBM.	(45)
00021	BALB/c	U87, U251, A172, and SHG44	P21, Notch1, Hes1, Hes5	Long intergenic noncoding RNA 00021 by epigenetically silencing p21 *via* the Notch pathway could promote GBM TMZ resistance.	LINC00021 expression is associated with the poor prognosis of GBM patients.	(46)
MIR22HG	Mouse	U87MG, LN229, LN1	Wnt/β-catenin, P21, P27, c-Muc, p-GSK3B, Cyclin-D1, LEF1	MIR22HG *via* suppressing the Wnt/β-catenin pathway could inhibit GBM progression.	MIR22HG expression is associated with poor prognosis in GBM	(12)
HOTAIR	Mouse	A172, LN229, A172/TMZ, LN229/TMZ	miR-519a-3p, RRM1, Vimentin, E-cadherin, CD63, N-cadherin, MAP3K1	Knockdown of HOTAIR by miR-519a-3p/RRM1 axis could regulate TMZ resistance.	–	(24)
HOTAIR	Human	U87, A172, U87/TMZ, A172/TMZ	miR-125, Cyt C, Caspase-3, HK2	HOTAIR by targeting miR-125 could promote chemoresistance in human GBM.	–	(25)
MIR155HG	Mouse, databases	A172, U251, A172/TMZ, U251/TMZ	Wnt/β-catenin, c-Myc, PTBP1, Cyclin-D1	Knockdown of MIR155HG by inhibiting the Wnt/β-catenin pathway *via* downregulation PTBP1 could increase glioma sensitivity to TMZ.	MIR155HG Upregulation was associated with poor prognosis	(13)
KCNQ1OT1 C	Mouse	U251, U87, U251/TMZ, U87/TMZ	miR-761, c-MYC, Pim-1L, p-MDR1, MDR1, Survivin	KCNQ1OT1 C by retrieving PIM1 From miR-761 could confer gliomas resistance to TMZ.	–	(47)
NCK1-AS1	Human	U251, A172, U251/TMZ, A172/TMZ	TRIM1, miR-137, TRIM24	NCK1-AS1 by modulating the miR-137/TRIM24 axis could increase the resistance of glioma cells to TMZ.	–	(48)
NCK1-AS1	Human	A172, LN229 A172/TMZ LN229/TMZ	miR-22-3p, IGF1R	NCK1-AS1 *via* miR-22-3p/IGF1R axis could enhance chemoresistance in glioma.	–	(49)
EPIC1	–	SNB19, T98G, U97MG, SNB19/TMZ, T98G/TMZ, U97MG/TMZ	Cdc20	Overexpression of EPIC1 *via* targeting Cdc20 could be useful in glioma treatment.	–	(50)
HOXD-AS1	TCGA dataset	U87, U251, U373, SNB19, U87/DDP, U251/DDP	miR-204, Caspase-3/9	Knockdown of HOXD-AS1 by buffering miR-204 could enhance cisplatin sensitivity.	high HOXD-AS1 expression had a poor prognosis	(51)
LINC01198	Human	U251, SNB-19, LN229, U87, U87/TMZ, U251/TMZ, LN229/TMZ, SNB-19/TMZ	PTEN, AKT, NEDD4-1	Overexpression of LINC01198 by enhancing the NEDD4-1-dependent repression of PTEN could promote glioma cell proliferation and resistance to TMZ.	LINC01198 high elevation was associated with a poor prognosis of glioma.	(52)
LINC00174	Human	U251, U87, U251/TMZ, U87/TMZ	SOX9, P13K/Akt	Knockdown of LINC00174 by regulating miR-138-5p/SOX9 axis could decrease chemoresistance to TMZ in glioma.	–	(53)
GAS5	–	U138, LN18 U87MG, U251MG, U138/Cis, LN18/Cis, U87/Cis, U251/Cis	mTOR, LC3I, LC3II, p-62	GAS5 by suppressing excessive autophagy in an mTOR‐dependent manner could facilitate glioma cell sensitivity to cisplatin.		(54)
CASC2	Human	U257, U87, U257/TMZ, U87/TMZ	mTOR, Beclin1, miR-193a-5p, LC3II/LCI	Upregulation of CASC2 through autophagy inhibition by buffering miR-193a-5p and regulating mTOR expression could sensitize glioma to TMZ cytotoxicity.	–	(55)
CASC2	Human	U251, U373, SNB19, U118, LN229 SNB19/TMZ, U251/TMZ	PTEN, AGO2, Akt, miR-181a	CASC2 by inhibiting miR-181a could increase sensitivity to TMZ in glioma.	CASC2 upregulation was associated with poor prognosis	(56)
CCAT2	Human	U251, U87, A172, SHG44	miR-424	CCAT2 by disturbing the normal function of miR-424 could enhance resistance in glioma.	CCAT2 upregulation was associated with a poor prognosis.	(57)
DANCR	Mouse	U87MG, LN18, U251MG, U138MG, U87MG/Cis, U251MG/Cis, U138MG/Cis	AXL, NF-kB, IkBa, PI3K/AKT	DANCR *via* activating AXL/PI3K/Akt/NF-κB signaling pathway could mediate cisplatin resistance in glioma cells.	–	(58)
MEG3	–	U87, U87/Cis	p-62, LC3 I/II, PARP	MEG3 by suppression of autophagy could enhance cisplatin resistance in glioma.	–	(30)
MSC-AS1	Human	LN229, HG-44 LN229/TMZ, SHG-44/TMZ	miR-373-3p, CPEB4, Bax, MCL-1, MRP-1, P-PIK3, Cyclin-D1, Caspase-3, PI3K/AKT	Knockdown of MSC−AS1 by regulating miR-373-3p/CPEB4 axis *via* PI3K/Akt pathway could inhibit cell growth and TMZ resistance in glioma.	MSC−AS1 upregulation was associated with a poor prognosis.	(50)
NR5A2	Mouse	U138, U251, A172, U87, U138/TMZ, U251/TMZ	NR5A2, PARP, NOTCH1, p21, Cyclin-D1, caspase-3, MMP2, E-cadherin	NR5A2 *via* regulating notch signal pathway could promote cell growth and resistance to TMZ in glioma.	NR5A2 overexpression was associated with the poor prognosis of glioma patients	(59)
ZFAS1	Human	U87, U251, NHA, A172, LN299, LN299/Cis, U251/Cis,	miR‐432‐5p	Knockdown of ZFAS1 by upregulating miR‐432‐5p could enhance cisplatin cytotoxicity in glioma.	Expression levels of ZFAS1 in clinical tissues is associated with poor prognosis	(60)
XIST	Human	LN229, U251, LN229/TZM, U251/TZM	Ago2, miR-29c	XIST *via* interacting with miR-29c and through DNA mismatch repair pathway could modulate the chemoresistance of glioma cell to TMZ.	Higher expression of XIST was associated with a lower OS rate.	(61)

## LncRNAs and Chemoresistance in Neuroblastoma

Wang et al. have demonstrated up-regulation of NORAD in neuroblastoma tissues and cell lines. Notably, NORAD expression has been inversely correlated with the survival of patients. NORAD has increased proliferation, metastatic ability, and resistance to doxorubicin while inhibiting apoptosis and autophagy in neuroblastoma cells through targeting miR-144-3p. HDAC8 has been identified as a direct target of miR-144-3p. NORAD up-regulation increases HDAC8 levels through suppression of miR-144-5p (53). SNHG7 is another lncRNA that modulates cisplatin-induced autophagy by regulating the miR-329-3p/MYO10 (62). Finally, SNHG16 regulates miR-338-3p/PLK4 axis to enhance cisplatin resistance in these cells (63). **Table 2** shows lncRNAs that modulate the response of neuroblastoma to chemotherapy.

**Table 2:** LncRNAs that modulate the response of neuroblastoma to chemotherapy.

LncRNA	Sample	Cell line	Target/pathway	Function	Kaplan Meier	Ref
NORAD	Human, Mouse	SK-N-SH, IMR-32, SK-N-SH/DOx, IMR-32/Dox	ATG5, LC3-I/II, P62, Beclin-1, PCNA, Cyclin-D1 Bcl-2, Bax, HDAC8	NORAD by upregulating HDAC8 *via* buffering miR-144-3p could enhance doxorubicin resistance of NB.	Higher expression of NORAD was associated with a lower OS rate.	(53)
SNHG7	Human	HUVEC, LAN-6, SK-N-AS, LAN-6/Cis, SK-N-AS/Cis	miR-329-3p, MYO10, LC3B-I/II, Beclin-1, P62	SNHG7 by regulating the miR-329-3p/MYO10 axis could enhance chemoresistance to cisplatin *via* modulating cisplatin-induced autophagy.	–	(62)
SNHG16	Human, Mouse	SK-N-AS, SK-N-SH, SK-N-AS/Cis, SK-N-SH/Cis	miR-338-3p, PLK4 MRP-1, p-gp, P13K/AKT	SNHG16 *via* regulating miR-338-3p/PLK4 axis could enhance cisplatin resistance in NB.	–	(63)

## Discussion

LncRNAs have acknowledged roles in the pathogenesis of tumors of the nervous system through various mechanisms including suppression of apoptotic pathways, induction of cell cycle progression, and enhancement of cell proliferation (10). A more clinically important aspect of lncRNA participation in the pathogenesis of nervous system tumors is their influence on the response of these neoplastic cells to chemotherapeutic agents. TMZ, cisplatin, and doxorubicin are the most important chemotherapeutic agents that are influenced by lncRNAs. Cancer stem cells are possibly the most critical cell population within the tumors which are affected by lncRNAs in this context. The competing endogenous RNA (ceRNA) function of lncRNAs has endowed them the aptitude to sequester miRNA, thus enhancing the expression of miRNA targets. MALAT1/miR-101, MALAT1/miR-203, TUSC7/miR-10a, NEAT1/let-7g-5p, AC003092.1/miR-195, SNHG15/miR-627, HOTAIR/miR-519a-3p, HOTAIR/miR-125, KCNQ1OT1/miR-761, NCK1-AS1/miR-137, NCK1-AS1/miR-22-3p and HOXD-AS1/miR-204 are among lncRNA/miRNA pairs that regulate resistance to chemotherapeutic agents in glioma/glioblastoma. SNHG7/miR-329-3p and SNHG16/miR338-3p have similar roles in neuroblastoma.

Based on the prominent effects of lncRNAs in the modulation of response of tumors of the nervous system to chemotherapeutic agents, prior knowledge about the levels of these transcripts in the tumor tissues would help in the design of appropriate therapeutic regimens. However, the particular locations of these tumors preclude invasive sampling. Therefore, peripheral blood/serum is an alternative tissue for this purpose. Consistent with this speculation, elevated serum levels of MALAT1 have been associated with the poor response of patients with glioblastoma to TMZ (17). However, most studies in this field rely on cell line experiments or animal studies without assessing the impact of circulating levels of lncRNAs in the long-term survival of patients. Moreover, the impact of genomic variants within lncRNAs in the modulation of response of glioblastoma/neuroblastoma to chemotherapeutic agents has not been assessed either in cell lines or in clinical settings. Such data would facilitate understanding the underlying mechanism of resistance to these agents and subsequently would pave the way for the design of therapeutic options to combat this phenotype.

Taken together, the contribution of lncRNAs in chemoresistance of glioma and neuroblastoma tumors has been assessed in independent studies. Yet, the role of these transcripts in the modulation of resistance to these agents has not been evaluated in other types of nervous system tumors. The proposed lncRNAs in this study are putative candidates for expression assays in other types of nervous system tumors.

## Author Contributions

SG-F and MT wrote the draft and revised it. HS, GS, AAg, AAb, and MMHT collected the data, designed the tables, and figures. All authors contributed to the article and approved the submitted version.

## Conflict of Interest

The authors declare that the research was conducted in the absence of any commercial or financial relationships that could be construed as a potential conflict of interest.
